# COVID-19 and Pregnancy: A Dangerous Mix for Bone Turnover and Metabolism Biomarkers in Placenta and Colostrum

**DOI:** 10.3390/jcm13072124

**Published:** 2024-04-06

**Authors:** Javier Diaz-Castro, Juan M. Toledano, Javier Sanchez-Romero, Africa Caño Aguilar, Estefanía Martín-Alvarez, Maria Puche-Juarez, Jorge Moreno-Fernandez, Maria Pinar-Gonzalez, Sonia Prados, María Paz Carrillo, Susana Ruiz-Duran, Catalina De Paco Matallana, Julio J. Ochoa

**Affiliations:** 1 Department of Physiology, Faculty of Pharmacy, Campus Universitario de Cartuja, University of Granada, 18071 Granada, Spain; javierdc@ugr.es (J.D.-C.); jjoh@ugr.es (J.J.O.); 2 Institute of Nutrition and Food Technology “José Mataix Verdú”, University of Granada, Biomedical Research Centre, Health Sciences Technological Park, Avenida del Conocimiento s/n, Armilla, 18071 Granada, Spain; 3 Instituto de Investigación Biosanitaria (IBS), 18012 Granada, Spain; 4 Nutrition and Food Sciences Ph.D. Program, University of Granada, 18071 Granada, Spain; 5 Department of Obstetrics and Gynecology, Hospital Clínico Universitario ‘Virgen de la Arrixaca’, El Palmar, 30120 Murcia, Spain; javier.sanchez14@um.es (J.S.-R.); katy.depaco@gmail.com (C.D.P.M.); 6 Institute for Biomedical Research of Murcia, IMIB-Arrixaca, El Palmar, 30120 Murcia, Spain; 7 Department of Obstetrics and Gynaecology, San Cecilio Universitary Hospital, 18071 Granada, Spain; 8 Unit of Neonatology, Pediatric Service, Hospital Universitario Materno-Infantil Virgen de las Nieves, 18014 Granada, Spain; 9 Department of Obstetrics & Gynaecology, Virgen de las Nieves University Hospital, 18014 Granada, Spain; mpazcb@gmail.com (M.P.C.);

**Keywords:** COVID-19, placenta, pregnancy, bone turnover, energy metabolism

## Abstract

**Background**: In pregnant women, COVID-19 can alter the metabolic environment, cell metabolism, and oxygen supply of trophoblastic cells and, therefore, have a negative influence on essential mechanisms of fetal development. The purpose of this study was to investigate, for the first time, the effects of COVID-19 infection during pregnancy with regard to the bone turnover and endocrine function of several metabolic biomarkers in colostrum and placenta. **Methods**: One hundred and twenty-four pregnant mothers were recruited from three hospitals between June 2020 and August 2021 and assigned to two groups: Control group and COVID-19 group. Metabolism biomarkers were addressed in placental tissue and colostrum. **Results**: Lipocalin-2 and resistin levels were higher in the placenta, revealing an underlying pro-inflammatory status in the gestation period for mothers suffering from COVID-19; a decrease in GLP-1 and leptin was also observed in this group. As for adiponectin, resistin, and insulin, their concentrations showed an increase; a decrease in GLP-1, leptin, and PYY was also reported in the colostrum of mothers suffering from COVID-19 compared with the control group. **Conclusions**: As for bone turnover, placental samples from mothers with COVID-19 showed lower levels of OPG, while DKK-1 increased compared with the control group. Colostrum samples showed higher levels of OPG, SOST, and PTH in the COVID-19 group, a fact that could have noteworthy implications for energy metabolism, fetal skeletal development, and postnatal bone density and mineralization. Further research is needed to explain the pathogenic mechanism of COVID-19 that may affect pregnancy, so as to assess the short-term and long-term outcomes in infants’ health.

## 1. Introduction

The first cases of a coronavirus-associated pneumonia were detected in December 2019 [1], and by March 2020, COVID-19 was declared a pandemic [2].

Available data indicate that pregnant women with COVID-19 pneumonia have a similar rate of admission to the intensive care unit (ICU) as nonpregnant women, but higher rates of preterm birth and cesarean section. Information on diseases associated with other highly pathogenic coronaviruses, such as SARS and MERS, provides insights into the effect of COVID-19 during pregnancy despite the limited data on the virus during pregnancy [3].

The placenta is an immune and endocrine organ composed of decidua that come from the mother’s endometrium and trophoblasts from the fetus [4]. During gestation, the placenta actively transports minerals and other nutrients to fetal blood, ensuring a correct energy supply and that the developing skeleton may accrete adequate mineral content [5]. The syncytiotrophoblasts contain neonatal Fc receptors (FcRn) that transport maternal IgG to the fetus, thereby conferring humoral immunity [6]. Viral infections during pregnancy have a broad spectrum of placental and neonatal pathology [7]. As part of the innate immune system, the syncytiotrophoblasts are a physical barrier to infections [8]. Recent research has shown that trophoblasts induce autophagy during viral infection and can also induce resistance to viruses in neighboring cells [9]. Vertical transmission of viruses during pregnancy is not completely understood [10]; however, during labor, maternal infections can spread to the newborn [11].

It is noteworthy that the extensive expression of angiotensin-converting enzyme 2 (ACE2) in the uteroplacental unit throughout gestation also supports a higher placental susceptibility to SARS-CoV-2 infection, potentially enabling vertical transmission. Another important aspect is the spatial–temporal distribution of ACE2 in the placenta during pregnancy. In early pregnancy, ACE2 is more concentrated in the decidua, in the area surrounding the villi, whereas a low content of ACE2 is found in the fetal–maternal interface. As gestation advances, the ACE2 protein expression pattern changes and higher expressions are found in syncytiotrophoblasts, villous endothelial cells, and cytotrophoblasts [12].

On the other hand, during the first days of lactation, colostrum is especially suited to the neonate due to its nutritional composition and in the non-nutritive bioactive factors that increase survival rate and adequate physiological development. Colostrum includes many beneficial bioactive factors. In contrast to infant formula, colostrum composition is dynamic, changing within a feeding and between mothers and populations [13]. During the postpartum period, the neonatal gastrointestinal tract undergoes several physiological changes which are profoundly influenced by colostrum ingestion because it contains large numbers of hormones, interleukins, and bioactive factors that modulate energy and bone metabolism [14]. As has been observed in placenta, breast milk is also influenced by COVID-19, with changes having been observed in the composition of macro and micronutrients of mothers suffering from the disease [15,16]. However, there are no studies related to metabolic processes, which is the object of this study.

In this context, bone remodeling and energy metabolism become two processes of great importance for the correct development of the newborn, in which, as mentioned previously, the placenta and breast milk play a great role. Moreover, there is a physiological link between the endocrine function of adipose tissue and bone turnover biofactors, which have effects on glucose and fatty acid metabolism, indicating that bone, in addition to being a support tissue, has an important endocrine role regulating energy metabolism and other metabolic pathways [17]. Both bone turnover [18] and energy metabolism [19] have been altered in people who have suffered COVID-19 infection. However, the possible alterations of these processes caused by the virus have never been studied in pregnant women—a situation which necessitates research, given the fact that the infection may not only have important repercussions on maternal health but also on fetal and neonatal development.

Taking into account the role of the placenta and colostrum on fetal and neonatal development, the aim of the current work includes investigations of clinical implications between placental function, colostrum feeding, and COVID-19 in order to elucidate their critical roles in neonate bone turnover and energy metabolism; these implications may have long-term effects not yet discovered in the newborn.

## 2. Materials and Methods

### 2.1. Subjects

One hundred and twenty-four pregnant mothers were recruited from three hospitals: “Hospital Materno-Infantil Virgen de las Nieves” (Granada, Spain), “Hospital Universitario Clínico San Cecilio” (Granada, Spain), and “Hospital Universitario Virgen de la Arrixaca” (Murcia, Spain) between June 2020 and August 2021. Figure S1 shows the flow diagram for subject recruitment and abandonment. Pregnant women without previous chronic pathologies were recruited in the corresponding consultation service and followed up on during the rest of the pregnancy. Inclusion criteria were: freely accepting to participate in the study, signing the informed consent, being pregnant with a regular ongoing pregnancy, having a body mass index of 18–30 kg/m^2^ in early pregnancy, and having suffered COVID-19 (PCR+, variant Alpha, B.1.1.7 from the 28th week onwards) for the COVID-19 group or not having suffered the infection (PCR- during the gestation period) for the control group. The exclusion criteria were: having a chronic disease that requires long-term treatment, an infectious disease, a body mass index of <18 or >30 kg/m^2^, malnutrition, chromosome/congenital malformations, fetal death, and non-acceptance of informed consent to participate in the study. The study was approved by the Bioethical Committee on Research involving human subjects (reference 10/20, date of 31 December 2020). Written informed consent was obtained from each participant after a detailed explanation of the study, and participants were free to withdraw from the study at any time in accordance with the Declaration of Helsinki.

Taking into account our main objective, and in accordance with the results obtained in a study of pregnant women in which some gestational parameters similar to the present study were determined (although not in mothers suffering from COVID-19, given the scarcity of studies in the scientific literature), a total of 55 mothers per group were needed—taking into account the loss of approximately 20% of the population during the study [20]. To increase the statistical power, while taking into consideration the prevalence of the infection in the Spanish population, we enrolled the following groups: Control Group (*n* = 61)—pregnant women who were not suffering from COVID-19 during pregnancy; and COVID-19 group (*n* = 63)—pregnant women who were suffering from COVID-19 during pregnancy (from the 28th week onwards). Throughout the delivery, clinical analytical estimators were controlled; simultaneously, the necessary samples were collected for subsequent analysis.

### 2.2. Placenta Sampling

The placenta’s size, shape, consistency, and completeness were assessed, as well as the presence of accessory lobes, placental infarcts, bleeding, tumors, and nodules. A cut was made to detach the umbilical cord from the placenta shortly after. The leftover umbilical tissues were then discarded after the umbilical artery was severed. A sample of placental cotyledons measuring 2 × 2 × 2 cm was also taken, omitting placental membranes. Samples were removed from the center of the placenta, avoiding necrosis, infarction, and calcification. The samples were then independently exposed to different washings with a chilled cold 0.9% NaCl solution with 0.1% butylated hydroxytoluene (BHT) (Sigma, St. Louis, MO, USA) and 1 mM Ethylenediaminetetraacetic corrosive (EDTA) (Sigma). Washings were repeated until no remaining blood was distinguished. The complete length of preparation time was under 15 min. Placenta fractions of homogenate were obtained from fresh placenta within the same reception day, and proteins were extracted from placenta samples through mechanical homogenization in tissue protein extraction reagent (T-PER) (Thermo Scientific Inc., Hanover Park, IL, USA), combined with a protease inhibitor cocktail (Sigma-Aldrich, St. Louis, MO, USA) at 4 °C, and stored at −80 °C for further analysis.

### 2.3. Multi-Elemental Analysis by Inductively Coupled Plasma-Mass Spectrometry (ICP-MS)

Prior to being placed in a resistant flask and dissolved in nitric acid, placenta samples were previously mineralized by a wet method in a sand bath (J.R. Selecta, Barcelona, Spain) and were then mixed with HNO_3_:HClO4 (69%:70%, *v*/*v*; Merck KGaA, Darmstadt, Germany; ratio 1:4, *v*/*v*) until all organic matter was completely removed. The following elements were measured with an Agilent 8800 ICP-MS: Ca, P, and Mg (Agilent, Santa Clara, CA, USA). After the proper dilution with 10% HNO_3_, all ICP-MS standards were made from ICP single-element standard solutions (Merck KGaA). Using rhodium as the internal standard, two sets of multi-element standards were created for calibration that included all of the relevant analytes at five different concentration levels.

### 2.4. Colostrum Sampling

Researchers met the mothers and educated them about the hand expression and electric pumping of colostrum every 2–3 h postpartum. Mothers were given instructions and pre-labeled sterile milk collection flasks to collect their colostrum—which was homogenized and frozen at −80 °C for the following analysis of energy and bone metabolism.

### 2.5. Energy and Bone Turnover Parameters

Ghrelin, Glucagon-like peptide 1 (GLP-1) (total), insulin, leptin, and gut hormone peptide YY (PYY) were determined using the HMHEMAG-34K Milliplex Human Metabolic Hormone Magnetic Bead Panel; Adrenocorticotropic hormone (ACTH), Dickkopf WNT signaling pathway inhibitor 1 (DKK-1), Osteoclacin (OC), Osteopontin (OPN), Osteoprotegerin (OPG), Sclerostin (SOST), and Parathormone (PTH) were determined in placenta protein extract and colostrum using the HMHEMAG-51K Milliplex Human Bone Magnetic Bead Panel; and Receptor Activator for Nuclear Factor κ B Ligand (RANKL) was determined using the HRNKLMAG-51K-01 Milliplex Human RANKL Magnetic Bead. Finally, adiponectin, adipsin, lipocalin-2/NGAL, and resistin were determined in placenta protein extract and colostrum using the HADK1MAG-61K MILLIPLEX MAP Human Adipokine Magnetic Bead Panel 1 (Millipore Corporation, Misuri, MO, USA). These parameters were all based on the immunoassays on the surface of fluorescent-coded beads (microspheres), following the specifications of the manufacturer (50 events per bead, 50 µL sample, gate settings: 8000–15,000, time out 60 s). The plate was read on a LABScan 100 analyzer (Luminex Corporation, Austin, TX, USA) with xPONENT software for data acquisition. Average values for each set of duplicate samples or standards were within 15% of the mean. Standard curve: GLP-1 (Total): 2.7–2000 pg mL^−1^, Insulin: 137–100,000 pg mL^−1^, Leptin: 137–100,000 pg mL^−1^, and PYY: 13.7–10,000 pg mL^−1^; ACTH: 1–6000 pg mL^−1^, DKK-1: 5–20,000 pg mL^−1^, OC: 146–600,000 pg mL^−1^, OPN: 98–400,000 pg mL^−1^, OPG: 7–30,000 pg mL^−1^, SOST: 24–100,000 pg mL^−1^, PTH: 5–20,000 pg mL^−1^, RANKL: 4.88–20,000 pg mL^−1^, adiponectin: 26–400,000 pg mL^−1^, adipsin: 12.8–200,000 pg mL^−1^, lipocalin-2/NGAL: 3.2–50,000 pg mL^−1^, and resistin: 6.4–100,000 pg mL^−1^. Analyte concentrations on placenta protein extracts and colostrum samples were determined by comparing the mean of the duplicate samples with the standard curve for each assay.

### 2.6. Statistical Analysis

Results are reported as mean values with their standard errors (SEM). Prior to any statistical analyses, all variables were checked for normality and homogeneous variance using Kolmogorov–Smirnoff’s and Levene’s tests, respectively. Differences between groups were tested using the non-parametric U-Mann–Whitney test when the responses were not normally distributed; or using *t*-test for independent samples when the responses were normally distributed. All statistical analyses were performed using SPSS software Version 27.0, 2020 (SPSS Inc., Chicago, IL, USA).

## 3. Results

All mothers were Caucasian Spaniards, and no differences were found at the moment of delivery in the age and parity of the mothers, gestational age, sex, and weight of the newborns, or maternal–fetal ejection (the strong involuntary reflex by the cervix in labor), revealing a term gestation with cephalic presentation and normal delivery. No statistically significant differences were found in the age, height, weight, and biochemical parameters of the volunteers participating in the study (Table S1).

**Figure 1:**
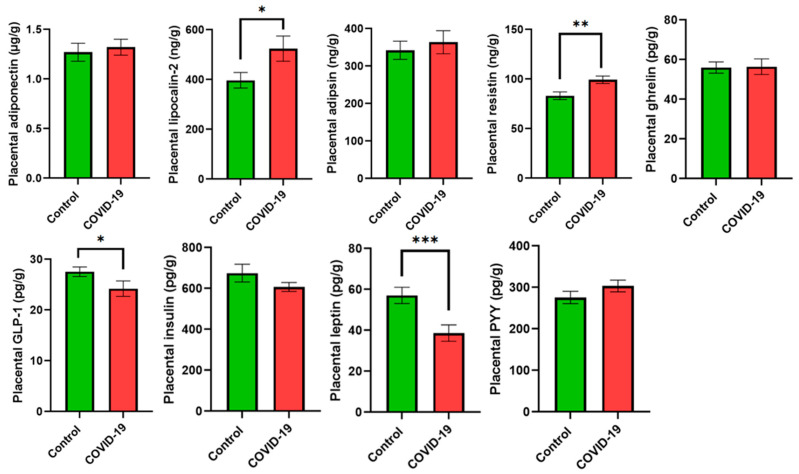
Energy metabolism biomarkers in placental samples. Data are shown as the mean values ± SEM. Significantly different from the control group (* *p* < 0.05, ** *p* < 0.01, *** *p* < 0.001).

**Figure 2:**
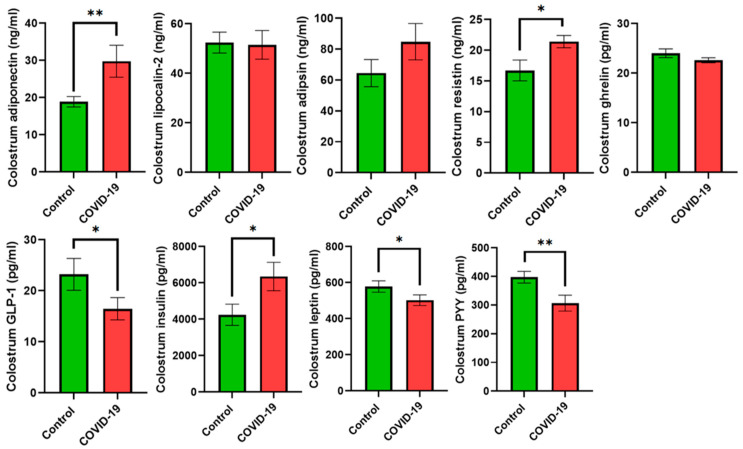
Energy metabolism biomarkers in colostrum samples. Data are shown as the mean values ± SEM. Significantly different from the control group (* *p* < 0.05, ** *p* < 0.01).

Energy metabolism biomarkers were addressed in placental tissue (Figure 1) and colostrum (Figure 2). Lipocalin-2 and resistin levels were higher in the placental tissue (*p* < 0.05 and *p* < 0.01, respectively) of mothers suffering from COVID-19 compared with the control group. On the other hand, GLP-1 and leptin were lower (*p* < 0.05 and *p* < 0.001, respectively) in the COVID-19 group compared with the control group. No statistically significant differences were observed for adiponectin, adipsin, ghrelin, insulin, or PYY. With regard to colostrum, adiponectin, resistin, and insulin concentrations showed an increase in the COVID-19 group (*p* < 0.01, *p* < 0.01, and *p* < 0.05, respectively) compared with the control group. On the other hand, a decrease in GLP-1 (*p* < 0.05), leptin (*p* < 0.05), and PYY (*p* < 0.01) was reported in the colostrum of mothers suffering from COVID-19 compared with the control group. Lipocalin-2 and ghrelin did not report any statistically significant differences.

**Figure 3:**
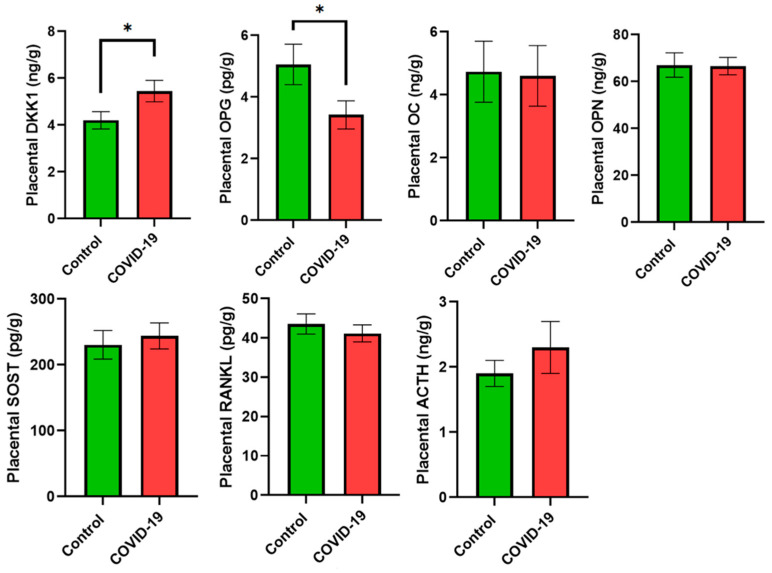
Bone turnover biomarkers in placental samples. Data are shown as the mean values ± SEM. Significantly different from the control group (* *p* < 0.05).

**Figure 4:**
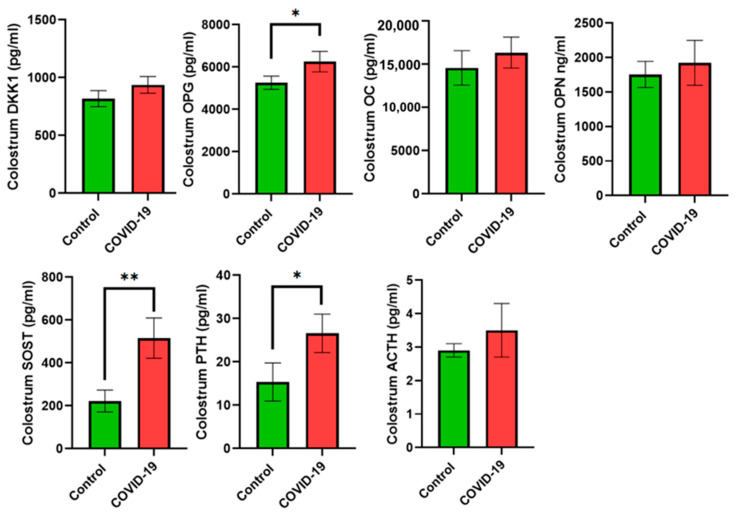
Bone turnover biomarkers in colostrum samples. Data are shown as the mean values ± SEM. Significantly different from the control group (* *p* < 0.05, ** *p* < 0.01).

As for bone turnover, placental samples from mothers with COVID-19 showed lower levels of OPG (*p* < 0.05), while DKK-1 increased compared with the control group (*p* < 0.05 for both). OC, OPN, SOST, RANKL, and ACTH did not show statistically significant differences between groups (Figure 3). Colostrum samples showed higher levels of OPG (*p* < 0.05), SOST (*p* < 0.01), and PTH (*p* < 0.05) in the COVID-19 group, while no statistically significant differences were observed for DKK-1, OC, OPN, and ACTH (Figure 4).

Additionally, placental concentrations of important minerals related to bone turnover have been evaluated, showing higher levels of phosphorus, calcium, and magnesium in the COVID-19 group (*p* < 0.001, *p* < 0.05, and *p* < 0.05, respectively) (Table 1).

## 4. Discussion

Intrauterine SARS-CoV-2 transmission is very rare because placenta may not express high levels of angiotensin-converting enzyme 2 (ACE2) and transmembrane serine protease 2 (TMPRSS2)—the proteins that facilitate SARS-CoV-2 entry into the trophoblasts. In addition, breastfeeding from a SARS-CoV-2-infected mother is safe for the neonate because SARS-CoV-2 has not been detected in breastmilk [21]. However, to date, there is no evidence in the scientific literature about the changes in placental function and colostrum biofactors caused by this infection in the mother that may have an influence on energy and bone turnover metabolism. In this sense, the primary finding of this study was the fact that SARS-CoV-2 infection induces several changes in bone turnover and energy metabolism in term placenta and colostrum, revealing that COVID-19 infection during pregnancy could induce homeostasis alterations affecting maternal health, fetus development, and postnatal life.

Our results are in agreement with those reported by Gupta et al. [22], in which SARS-CoV-2 increased lipocalin-2 levels in the general population. Lipocalin-2 is a neutrophil-associated protein that has a key role in immune function, and it is linked to cardiovascular and renal diseases [23]. The increased levels of lipocalin-2 interact with matrix metalloproteinases and other inflammatory mediators, indicating a crucial role in vascular inflammation [24], representing an independent thrombotic risk factor and thromboembolic complications [25]. This result, together with our previous findings reporting an up-regulation of placental ferritin expression in women who suffered SARS-CoV-2 infection compared with healthy women [26], could be the explanation as to why many women suffering from COVID-19 feature a pro-coagulant state [27] that would finally lead to over-activation of coagulation and thromboembolic complications in the placenta blood vessels.

Resistin was also measured in the current study, showing—for the first time in the literature that we had studied—that mothers who suffered COVID-19 had increased levels of resistin in their placenta and colostrum. It has been previously reported that resistin levels were higher in patients suffering COVID-19 [28]; it is also known that resistin enhances the NF-kB transcription factors leading to higher expression of pro-inflammatory cytokines and cytokine storms in COVID-19 [29]. Additionally, resistin responds to hyperglycemia and metabolic stress, which has a potential role in regulation of placental mitochondrial abundance and function [30].

Leptin was lower in the placenta and colostrum of mothers who suffered COVID-19, and this could have important implications for fetal lung development because this protein hormone modulates fetal respiratory health through pleiotropic actions [31]. Leptin has a key role in lung development and maturation of fetal lungs because it is involved in surfactant protein production by fetal type II pneumocytes [32]. In addition, leptin regulates bronchial diameter by counteracting the parasympathetic effect on the airways [33]. It is also important for immune system development and function because leptin-deficient subjects show increased risk of death due to infections [34]. Low leptin production is linked with the pathogenesis of several pulmonary diseases and pulmonary arterial hypertension. In patients with pneumonia, leptin is inversely correlated with biomarkers of inflammation [31], a result that could be correlated with the pro-inflammatory state in mothers suffering from COVID-19 [26].

GLP-1 was also lower in the placenta and colostrum of mothers who suffered COVID-19. Activation of the GLP-1 receptor not only exerts glycemic control but also regulates plenty of pathophysiological processes because its activation inhibits cytokine release [35]. Taking into account its physiological role, we hypothesize that reduced placental and colostrum levels of GLP-1 could be a consequence of the development and progression of the inflammatory signaling in mothers who suffered COVID-19.

Another important hormone in glucose metabolism is insulin. In our study, insulin was higher in the colostrum of mothers suffering from COVID-19. It is well known that inflammation and oxidative stress impair insulin activity with consequent insulin resistance [36]; therefore, this could be a compensatory mechanism to cope with the altered insulin signaling due to the increased oxidative stress and inflammation recorded in mothers suffering from COVID-19 [26].

On the other hand, PYY was reported to have a trophic effect in the duodenum of a lactating rat, but no effect was observed in the adult rat. In addition, PYY increased the weight and size of the duodenum, ileum, and colon of adult mice [37]; therefore, the reduction of this hormone, as recorded in the colostrum of mothers who suffered COVID-19, could have implications for the development of both the fetal and newborn gastrointestinal tract.

Finally, an adipokine of great importance in energy metabolism is adiponectin, which avoids endothelial apoptosis and has the ability to neutralize oxidative stress and its deleterious effects because adiponectin decreases the production of ROS in endothelial cells to enhance vascular function [38]; therefore, we hypothesize that the increase recorded in placenta could be a compensatory mechanism to cope with the increased oxidative stress in mothers suffering from COVID-19 [26].

Along with energy metabolism, bone turnover has also been affected in mothers suffering from COVID-19 disease. OPG is an inhibitor of RANKL, which enhances bone formation, protecting against bone loss. In our study, placenta OPG levels decreased, and in this regard, it has previously been reported that other viruses also affect bone turnover by inducing the production of pro-inflammatory cytokine levels that, in turn, dysregulate the RANKL–OPG axis, augmenting bone loss [39]. In a previous study [26], it was reported that SARS-CoV-2 induced an over-production of reactive oxygen species (ROS) and a reduction in the antioxidant system of the placenta, inducing cell damage to the trophoblasts’ bioconstituents (proteins, lipids, and DNA). In this sense, ROS act as second messengers, inducing inflammasome activation, DNA damage, cell cycle arrest, and apoptosis; ROS have also been linked to osteoporosis [40,41]. In addition, oxidative stress has been linked to bone loss through the dysregulation of OPG expression favoring osteoclastogenesis via the reduction of OPG [42]. In addition, due to the key role of OPG in bone formation, we hypothesize that its increased levels in colostrum could be a compensatory mechanism to cope with the impaired fetal bone homeostasis due to the impairment of bone turnover biomarkers recorded in the placenta of mothers suffering from COVID-19. This compensatory mechanism, however, does not seem effective in improving bone homeostasis in the newborn because SOST increased in colostrum; this glycoprotein also affects bone resorption by up-regulating pro-osteoclastogenic signaling between osteocytes and osteoclasts [43], resulting in decreased bone formation, mass, and strength.

PTH was higher in the colostrum of mothers who suffered COVID-19, and the mechanism explaining this increase in PTH levels can be explained by several factors: firstly, parathyroid gland function could be impaired during inflammatory response due to the exacerbated inflammatory signaling, diminishing the liberation of PTH [44]; also, previous studies conducted during severe acute respiratory syndrome demonstrated the presence of viruses in the parathyroid gland, impairing its endocrine function [45,46]. Moreover, a hypothesis has also been previously reported of a suboptimal compensatory PTH response secondary to the induced parathyroid gland alteration in COVID-19 patients [47]. Therefore, this increase in PTH levels, as recorded in the colostrum of mothers who suffered COVID-19, could also affect the neonate’s bone turnover process.

In addition, we found an increase in calcium, phosphorus, and magnesium in the placenta of mothers who suffered COVID-19, revealing an accumulation of these minerals due to the polarity of the syncytiotrophoblast structure, which implies particularities in the transfer of minerals through the placenta [48]. Under physiological conditions, the concentration of these elements is low in the cytosol of syncytiotrophoblast cells in order to favor the fetal transfer [49]. Therefore, these increased levels of calcium, phosphorus, and magnesium found in the placenta reveal an impairment in the maternal–fetal transfer mineral flow, highlighting once more the impairment in the fetal bone mineralization process during gestation.

DKK-1 is a pro-inflammatory immunomodulator widely expressed in many tissues, including bone, skin, placenta, and endothelium [50]. Enhanced DKK-1 expression has been reported within advanced carotid plaques, indicating DKK-1 as an important modulator of platelet-mediated endothelial cell interaction [51]. In addition, DKK-1 plays a pro-inflammatory role for human vascular endothelial cells to secrete IL-6 and IL-8 during infections [52]. Once more, this result, along with our previous findings reporting an up-regulation of ferritin expression during COVID-19 [26], could explain the increased pro-coagulant state of pregnant women suffering from COVID-19 [27]. On the other hand, DKK-1 has a key role on fetal and postnatal bone development and bone health and disease. DKK-1 has a central role in skeletal development and homeostasis because it inhibits osteoblastogenesis [53], a fact that could have noteworthy implications for fetal skeletal development. DKK-1 also induces the formation of adipocytes [54]; therefore, its lower levels in placenta could also be linked with the lower leptin levels recorded in mothers who suffered COVID-19 in the current study.

In the current study, we found some limitations or aspects that should be considered in future studies. For example, since the study participants represented a highly heterogeneous group, a larger sample size would have been desirable, as this would have led to more solid evidence and likely more statistically significant differences. It also would have been desirable to study the biomarkers in neonates in order to understand the full extent to which the observed maternal and placental outcomes may affect the newborn because a possible effect of the alterations observed in placenta could induce some clinical outcomes in the newborn. Finally, a subsequent monitoring of subjects would have provided more information about the evolution of the disease and possible non-immediate complications derived from it.

The results obtained in the current study show several clinical manifestations of COVID-19 that could affect the offspring, including impairment of fetal skeletal development, postnatal bone density and mineralization, and metabolic endocrine function of the placenta, together with the changes of colostrum composition that influence the development of the neonate.

## 5. Conclusions

In conclusion, our results suggest, for the first time, that COVID-19 affects energy metabolism and bone turnover biomarker homeostasis in placenta and colostrum. Lipocalin-2 and resistin levels were higher in the placenta, revealing an underlying pro-inflammatory status in the gestation period for mothers suffering from COVID-19. With regard to colostrum, adiponectin, resistin, and insulin concentrations showed an increase, while a decrease in GLP-1, leptin, and PYY was also reported in the colostrum of mothers suffering from COVID-19 compared with the control group. In relation to bone turnover, the mothers who suffered COVID-19 featured higher levels of DKK-1, calcium, phosphorus, and magnesium as well as lower levels of OPG in placenta and higher levels of OPG, SOST, and PTH in colostrum—findings that would have noteworthy implications for fetal skeletal development and postnatal bone density and mineralization. Due to the key roles of metabolic endocrine function, bone turnover in placental function, and the profound influence of colostrum in the development of the neonate, further research is needed to elucidate the pathological mechanisms and repercussions of COVID-19 that affect gestation and breastfeeding as well as the short-term and long-term consequences in the mothers’ and newborns’ health.

## Figures and Tables

**Table 1:** Placental levels of minerals related to bone turnover.

	Control	COVID-19
Calcium (mg/g DM)	2.01 ± 0.26	3.02 ± 0.51 *
Phosphorus (mg/g DM)	9.34 ± 0.42	11.78 ± 0.63 **
Magnesium (mg/g DM)	0.32 ± 0.07	0.38 ± 0.11 *

Data are shown as the mean values ± SEM. Significantly different from the control group (* *p* < 0.05, ** *p* < 0.01). DM = dry matter.

## Data Availability

All of the data are contained within the article.

## Supplementary Materials

The following supporting information can be downloaded at: https://www.mdpi.com/article/10.3390/jcm13072124/s1; Figure S1: Flow chart showing the progress and abandonment of study subjects; Table S1: Clinical and sociodemographic characteristics of healthy mothers and those who have suffered COVID-19.
