# Supplementary material for: COVID-19 and Pregnancy: A Dangerous Mix for Bone Turnover and Metabolism Biomarkers in Placenta and Colostrum
Source: J Clin Med. 2024 Apr 6;13(7):2124. doi: 10.3390/jcm13072124 (PMC11012405; doi:10.3390/jcm13072124)
Supplement: Supplementary file 1 [file jcm-13-02124-s001.zip › jcm-2903655-supplementary.pdf]

Supplementary Materials

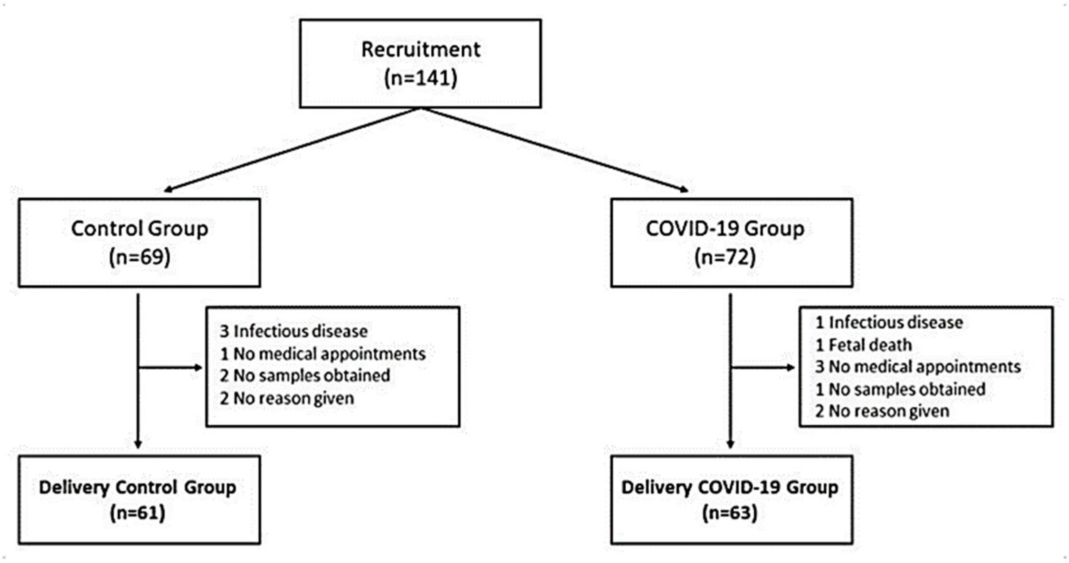

Figure S1: Flow chart showing the progress and abandonment of study subjects.

Table S1. Clinical and sociodemographic characteristics of healthy mothers and those who have suffered COVID-19.

	Control group		Covid-19 group
Age (years)	31.58 ± 1.09		31.96 ± 0.78
Weight (kg)	73.69 ± 2.55		74.12 ± 2.67
Height (cm)	166.83 ± 1.03		163.97 ± 0.68
BMI (kg/m^2)	26.31 ± 1		27.3 ± 0.8
Parity	Uni (%):	52.22	53.14
	Multi (%):	47.28	46.86
Delivery method	V (%):	56.2	58.7
	A (%):	21.8	19.9
	C (%):	21.8	23.8
Hemoglobin 2nd T (g/L)	11.88 ± 0.23		11.53 ± 0.13
Hemoglobin 3rd T (g/L)	11.96 ± 0.23		11.72 ± 0.17
Hematocrit 2nd T (%)	35.32 ± 0.61		34.11 ± 0.37
Hematocrit 3rd T (%)	35.70 ± 0.63		34.82 ± 0.47
Serum Iron 3rd T (µg/dL)	97.05 ± 14.35		60.14 ± 9.87**
Total Cholesterol 3rd T (mg/dL)	205.29 ± 11.23		208.13 ± 14.05
AST 3rd T (IU/L)	29.01 ± 12.10		21.01 ± 3.21
ALT 3rd T (IU/L)	22.15 ± 10.24		18.17 ± 4.11

BMI: Body mass Index; T: Term; V: Vaginal; A: Assisted Vaginal Delivery; C: Cesarean; AST: aspartate aminotransferase; ALT alanine aminotransferase. **Significantly different from the control group ($P < 0.01$, Student's t test).
